# Microscopic Chemical Reaction Mechanism and Kinetic Model of Al/PTFE

**DOI:** 10.3390/polym16111467

**Published:** 2024-05-22

**Authors:** Mengmeng Guo, Xiangrong Li, Yongkang Chen, Haifu Wang

**Affiliations:** 1 State Key Labratory of Explosive Science and Technology, Beijing Institute of Technology, Beijing 100081, China; guomengmeng0902@163.com; 2 Department of Weapons and Control, Army Armored Corps Academy, Beijing 100072, China; lxr118@163.com

**Keywords:** Al/PTFE reactive material, reactive force field (ReaxFF), interface reaction, oxide layer, mechanism function

## Abstract

In order to study the microscopic reaction mechanism and kinetic model of Al/PTFE, a reactive force field (ReaxFF) was used to simulate the interface model of the Al/PTFE system with different oxide layer thicknesses (0 Å, 5 Å, 10 Å), and the thermochemical behavior of Al/PTFE at different heating rates was analyzed by simultaneous thermal analysis (TG-DSC). The results show that the thickness of the oxide layer has a significant effect on the reaction process of Al/PTFE. In the system with an oxide layer thickness of 5 Å, the compactness of the oxide layer changes due to thermal rearrangement, resulting in the diffusion of reactants (fluorine-containing substances) through the oxide layer into the Al core. The reaction mainly occurs between the oxide layer and the Al core. For the 10 Å oxide layer, the reaction only exists outside the interface of the oxide layer. With the movement of the oxygen ions in the oxide layer and the Al atoms in the Al core, the oxide layer moves to the Al core, which makes the reaction continue. By analyzing the reaction process of Al/PTFE, the mechanism function of Al/PTFE was obtained by combining the shrinkage volume model (R3 model) and the three-dimensional diffusion (D3 model). In addition, the activation energy of Al/PTFE was 258.8 kJ/mol and the pre-exponential factor was 2.495 × 10^15^ min^−1^. The research results have important theoretical significance and reference value for the in-depth understanding of the microscopic chemical reaction mechanism and the quantitative study of macroscopic energy release of Al/PTFE reactive materials.

## 1. Introduction

Reactive destructing materials (RMs) represent a novel class of materials characterized by maintaining stability under normal conditions while reacting upon impact [1]. When kill elements, prepared from RMs, strike a target at high speed, they generate a dual-destructive mechanism of penetration and explosion. RMs have been widely used in fragments, liners, and other kill elements [2,3]. Compared to traditional kill elements, reactive fragments exhibit larger kill radii and superior capability to ignite aviation kerosene. Similarly, liners made from RMs significantly enhance the destructive effects on concrete targets. RMs can be broadly categorized into four types: fluoropolymer-based, metal-intermetallic compounds, alloys, and amorphous materials. Among these, the fluoropolymer-based system is composed mainly of polytetrafluoroethylene (PTFE) powder and active metal powder, with Al/PTFE being the most representative [4]. 

Under high-velocity impact or explosive loads, Al/PTFE releases substantial chemical energy. Researchers have delved into the energy release mechanisms of Al/PTFE under high strain rate loading and identified a two-step process: first, a temperature rise due to high strain rate loading, followed by a chemical reaction triggered by the elevated temperature. Yang [5] employed a meso-scale model to study the temperature rise of Al/PTFE under impact loads, indicating that higher impact velocities lead to greater temperature increases. Cai [6] analyzed the effect of material porosity on the temperature rise of Al/PTFE, suggesting that collapsing and closing of internal pores under impact loads lead to rapid temperature increases and hotspot formation, thereby initiating reactions. Lu [7] proposed a theoretical model to describe the behavior of impact-induced chemical reactions: under impact, hotspots are formed in the PTFE matrix. When the temperature of these hotspots reaches the decomposition temperature of PTFE, CF_2_ gas is released. At the same time, local fragmentation of the PTFE matrix brings Al particles into contact with CF_2_ gas, initiating subsequent chemical reactions. Some researchers [8] believe that after the temperature rise induced by impact, the oxide layer ruptures under the stress from the melting and expansion of the aluminum core, resulting in an eruption-like effect on the aluminum particle surfaces and rapid reactions with oxides. Regarding specific reaction mechanisms, Losada [9] studied the reaction between PTFE monomer C_2_F_4_ and Al under anaerobic/aerobic conditions using quantum mechanics, identifying intermediate products and final products. Jun [10] employed molecular dynamics to simulate the pyrolysis of PTFE and the reactions between pyrolysis products and Al, indicating the CF_3_ + Al/CF_2_ + AlF reaction pathway as the most feasible. However, these studies did not consider the oxide layer surrounding the aluminum particles. Overall, while temperature rise is a necessary condition for reactions to occur, the reaction process of Al/PTFE under temperature remains unclear, especially considering the oxide layer’s role.

Numerical simulation is an essential tool for studying the energy release process of Al/PTFE. To accurately simulate the energy release process of Al/PTFE, a model that can accurately describe the reaction is necessary. Rosencrantz [11] and Ren [12] utilized the Lee–Tarver ignition and growth model to calculate the reaction rate of Al/PTFE, but its complex parameterization poses challenges in controlling the reaction rate. Currently, reaction kinetics models, such as the Avrami–Erofeev equation with *n* = 3/2 used by Zhou [13] and *n* = 0.625 by Zheng [14], or the Jander model [15], are employed to calculate the reaction process of Al/PTFE. However, uncertainties remain in the kinetics models and reaction mechanism function of Al/PTFE.

This study employs a reliable reactive force field to simulate the reaction process of Al/PTFE. Thermal analysis experiments are conducted to analyze Al/PTFE, revealing the chemical reaction mechanisms of the Al/PTFE system with an oxide layer. A kinetic model for the Al/PTFE reactive material is established based on these findings. The results of this research hold significant reference value for a deeper understanding of the microchemical reaction mechanisms of reactive materials with oxide layers. Moreover, they are of crucial importance for the development of numerical calculation methods for the impact energy release of reactive materials.

## 2. Experiments

### 2.1. Experimental Method

**Figure 1:**
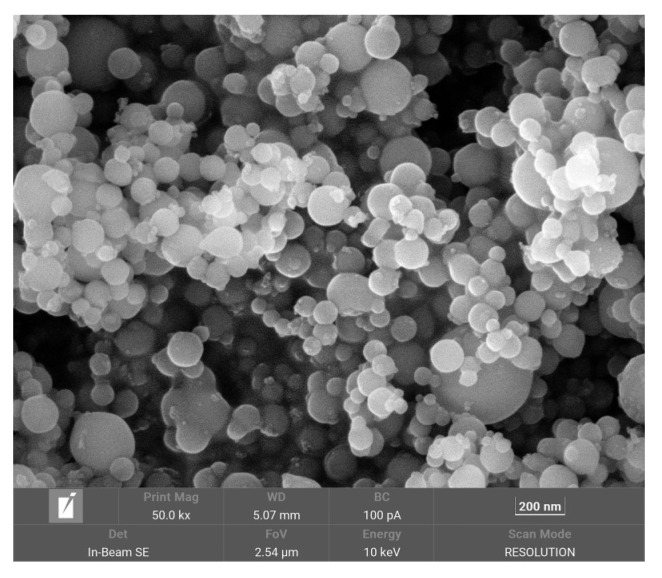
SEM photo of nano-Al powder.

Al/PTFE with an Al particle size of 100 nm was prepared by the wet mixing method, as shown in Figure 1. PTFE powder and nano-sized Al powder were mixed at a mass ratio of 76.5 to 23.5, and anhydrous ethanol was used as the medium. The magnetic stirrer was used to wet the mixture in a vacuum environment to obtain a uniformly mixed material. After drying in a vacuum drying oven, an Al/PTFE sample powder was obtained. The non-isothermal thermochemical analysis of Al/PTFE reactive materials was conducted by simultaneous thermal analysis (TG-DSC). The temperature could be controlled in the range of 30–800 °C by using a HITACHI STA200 simultaneous thermal analyzer. The experiment was conducted in argon atmosphere at four heating rates of 5 K/min, 10 K/min, 15 K/min, and 20 K/min. The specific conditions are shown in Table 1.

### 2.2. Experimental Results

**Figure 2:**
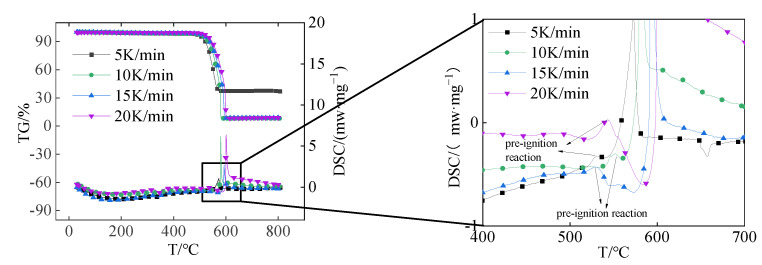
TG-DSC thermal analysis curve.

Figure 2 shows the TG-DSC thermal analysis curves of Al/PTFE at different heating rates. It can be seen from the diagram that the TG and DSC curves at different heating rates have similar trends. The TG curve remained flat before 500 °C, there was obvious mass loss between 500 °C and 600 °C, and there was only one mass loss stage in the whole reaction process. The DSC curve shows an endothermic peak at about 344 °C, which is near the melting point of PTFE [16] and is caused by the melting of the PTFE matrix, and an obvious exothermic peak at about 600 °C. It is worth noting that before the main exothermic peak appears, DSC curves of different heating rates have a small exothermic peak, and the reaction of the small exothermic peak is a pre-ignition reaction. The exothermic peak near 600 °C is caused by the reaction of Al/PTFE. The temperature at the beginning of this peak is defined as the initial reaction temperature (*T*_i_), the peak maximum temperature is defined as the peak reaction temperature (*T*_p_), and the temperature at the end of the peak is defined as the final reaction temperature (*T*_f_). Table 2 lists the characteristic temperature (*T*_i_, *T*_p_, *T*_f_) under different heating rates. The characteristic temperature is different with different heating rates. As the heating rate increases, the thermal effect per unit time increases, the thermal inertia increases, and the temperature difference increases, so that the reaction’s exothermic peak and other temperature characteristics move toward the high-temperature direction. It can be seen from the TG curve that the final mass loss is about 63% when the heating rate is 5 K/min, and the melting endothermic peak of Al appears in the DSC curve at 657 °C. It indicates that there are incompletely reacted Al particles at a heating rate of 5 K/min. In the thermal analysis experiments of other heating rates, the TG curve will change from slow decline to sudden decline, and the final mass loss is about 92%, which indicates that all the samples reacted.

## 3. Molecular Dynamics Simulation

### 3.1. Simulation Method

The Al/PTFE molecular dynamics simulation was carried out using the ReaxFF reaction force field released by the Van Duin team, which can describe the interaction of the four elements in C-F-Al-O [17]. The ReaxFF is an empirical force field based on quantum mechanics and experiments [18,19]. It relies on the bond order to simulate the generation and breaking of bonds. The force field decomposes the system energy into the following items:

E_system_ = E_bond_ + E_over_+ E_val_ +E_tors_ +E_vdWals_+ E_coulomb_+ E_specifIc_

where E_bond_, E_val_, and E_tors_ are, respectively, bond energy, valence angle energy, and torsion angle energy. E_over_ is the correction energy caused by over-coordination, and E_vdWals_ is the energy provided by the Van der Waals force. E_coulomb_ is the Coulomb energy, and E_specifIc_ is the specific energy in the system such as hydrogen bond and lone electron pair. In particular, ReaxFF enables simulations involving reactive events at the interface between solid, liquid, and gas phases, which is made possible because the ReaxFF description of each element is transferable across phases [20]. Researchers including Senftle [21] and Liu [22] have successfully studied the reactions at the interface using the ReaxFF.

**Figure 3:**
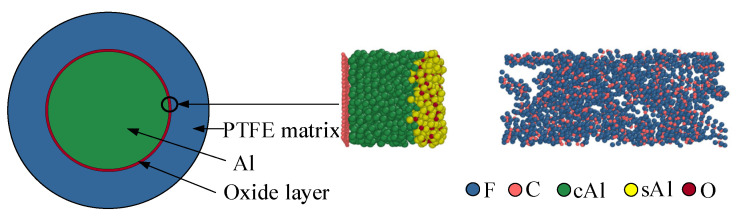
Molecular dynamics initial structure.

Al has a high affinity for oxygen. After Al particles are exposed to air, the outer layer of Al atoms is oxidized by oxygen atoms in the environment to form a dense Al_2_O_3_ layer. Therefore, the Al particle structure in Al/PTFE is usually a core/shell structure with Al as the core and the Al_2_O_3_ oxide layer as the shell. Based on the above analysis, an interface model is established, as shown in Figure 3. To protect the bottom surface of the Al slab, a single graphene layer is added [17]. The carbon chain length of each PTFE molecule is twenty. In the model, sAl (shellAl, yellow) represents Al in the oxide layer, and cAl (coreAl, green) represents Al in the Al core. The fluorine atom is represented by blue, the oxygen atom is represented by red, and the carbon atom is represented by pink.

**Figure 4:**
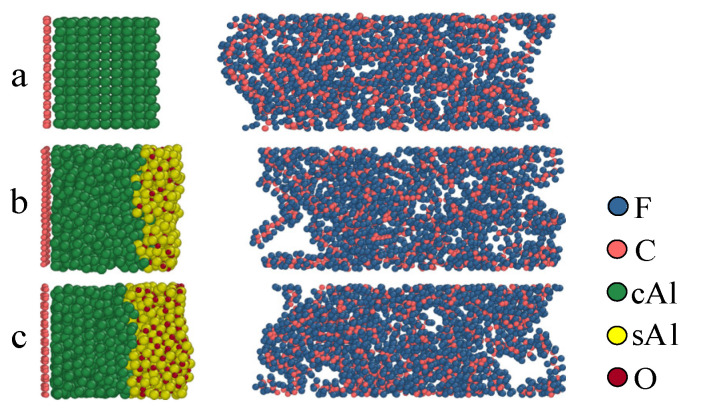
Initial structure at different oxide layers. (**a**) 0 Å; (**b**) 5 Å; (**c**) 10 Å.

The particle size of aluminum significantly influences the reactions of Al/PTFE. Different particle sizes result in distinct DSC curves [15]. Under impact, the ignition threshold and relaxation time also vary noticeably [23]. This is due to the different thicknesses of the oxide layer [24] and contact areas at varying particle sizes. In order to study the effect of oxide layer size on the reaction in the Al/PTFE chemical reaction process, the models of oxide layers with different thicknesses were established. The model was relaxed using Lammps to obtain a reasonable initial structure [25]. In reality, the oxide layer outside aluminum particles is thicker. Considering computational efficiency and cost, the thickness of the oxide layer after relaxation at a simulated temperature of 300 K is 0 Å in Figure 4a, 5 Å in Figure 4b, and 10 Å in Figure 4c. In different Al/PTFE model structures, the number of cAl remains the same when only the thickness of the oxide layer is changed.

The reactive molecular dynamics simulation was performed using Lammps. The initial temperature of the Al/PTFE simulation system with three different structures was set to 1200 K–1900 K (1200 K, 1500 K, 1750 K, 1900 K). The specific simulation conditions are shown in Table 3. In the simulation process, the temperature is controlled by the Berendson thermostat, and the dynamics are simulated by the NVT canonical ensemble. The MD time step length of the simulation is 0.1 fs.

### 3.2. Simulation Results

**Figure 5:**
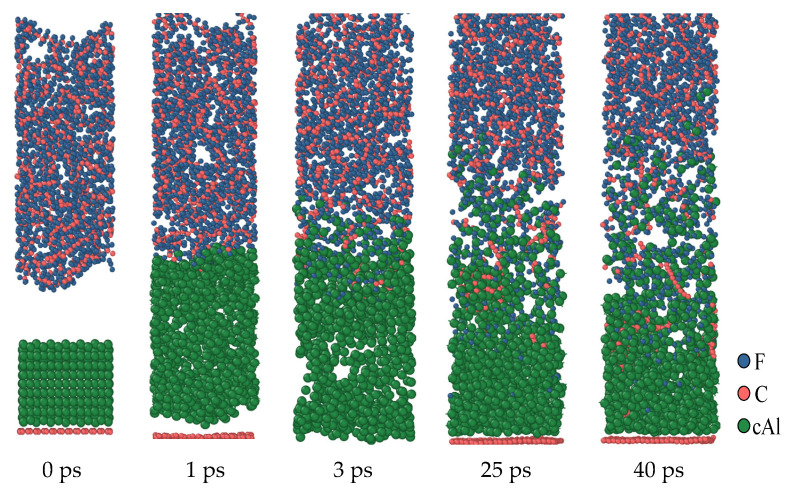
Al/PTFE reactive process of the oxide layer at 0 Å.

Figure 5 shows the reaction process of the first structure (a, 0 Å) at a simulated temperature of 1900 K. Under the influence of thermal expansion, when the simulation is conducted to 1 ps, Al contacts with PTFE and reacts immediately to form an AlF_x_ product layer. The formation of this product layer makes it impossible for Al to directly react with PTFE or PTFE products, and it needs to react with PTFE decomposition products through the product layer. As time goes on, this product layer continues to increase. The dynamics simulation results show that at the simulation temperature of 1900 K, some C atoms from PTFE will deposit on the surface of Al to form Al-C clusters. At the same time, some C atoms exist in the form of simple substances, which indicates that the behavior of C atoms in the reaction process is complex under high-temperature conditions.

**Figure 6:**
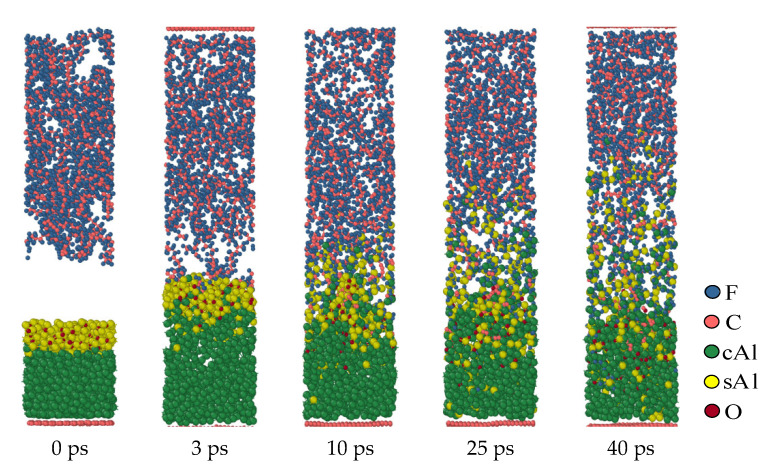
Al/PTFE reactive process of the oxide layer at 5 Å.

Figure 6 shows the dynamics process of the second structure (b layer, 5 Å) at the simulated temperature of 1900 K. Although structure (b) has an oxide layer, under high-temperature conditions, the oxide layer is affected by thermal rearrangement. Its compactness is changed, and it can even be considered that the oxide layer is broken. This makes the oxide layer lose its original hindering effect. Therefore, the reaction process of the structure is like that of the first structure (a), and the fluorine-containing substance diffuses through the product layer to react with Al. In the second structure (b), similar to the first structure (a), Al contacts with PTFE at high temperatures and reacts immediately to form an AlF_x_ product layer. The formation of this product layer makes it impossible for Al to directly react with PTFE or PTFE products, and it is necessary to react with PTFE decomposition products through the product layer. As time goes on, this product layer continues to increase.

**Figure 7:**
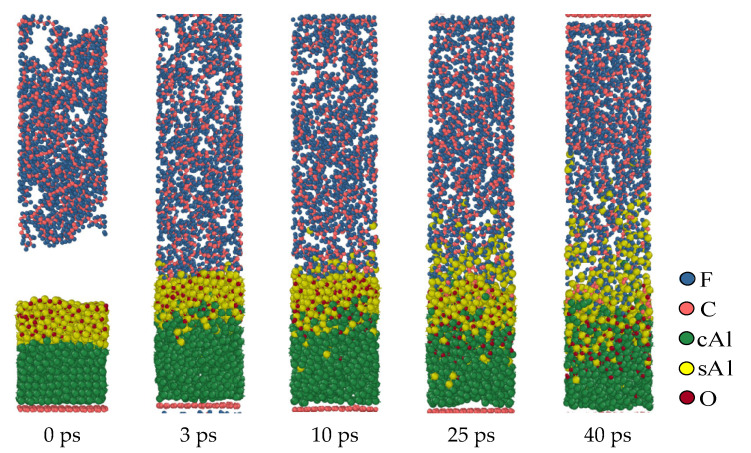
Al/PTFE reactive process of the oxide layer at 10 Å.

Figure 7 shows the molecular dynamics evolution process at a simulated temperature of 1900 K when the oxide layer thickness is 10 Å (structure c). In the simulation results, the reaction in structure (c) only occurs outside the oxide layer, and the oxide layer is not broken during the full process. Different from the oxide layers with thinner structures, when the thickness of the oxide layer is 10 Å, the fluorine-containing material cannot diffuse through the oxide layer into the interior of the Al core during the reaction. This means that in the third structure (c), the reaction is still confined to the surface of the oxide layer and cannot penetrate the oxide layer into the interior of the Al core. The oxide layer remains intact during this process and hinders the diffusion of fluorine-containing substances, preventing them from entering the Al core for reaction.

## 4. Discussion

### 4.1. Reaction Process

**Figure 8:**
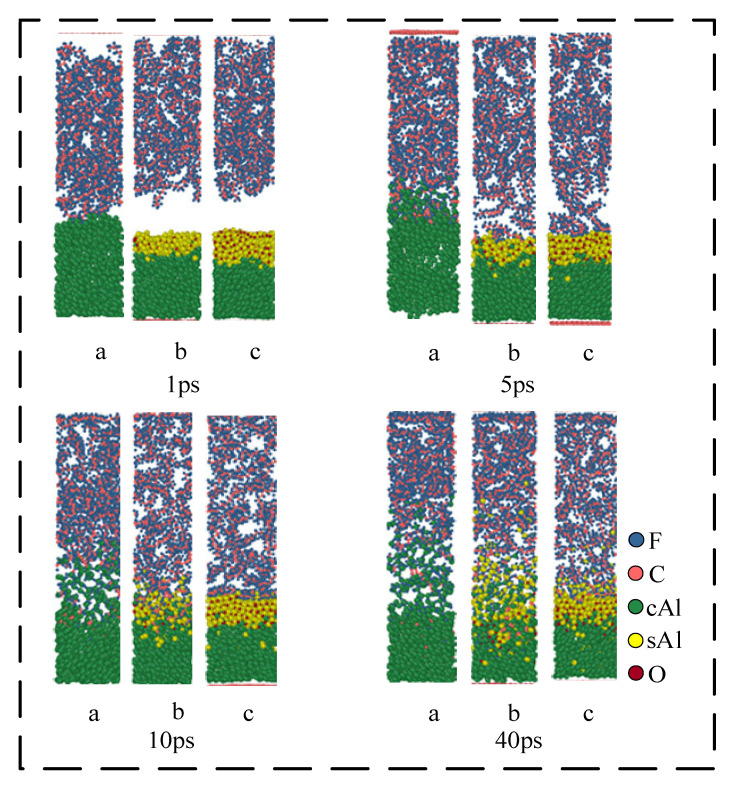
Reaction process of Al/PTFE system. (a) 0 Å; (b) 5 Å; (c) 10 Å.

Figure 8 shows the reaction process of the Al/PTFE system with different oxide thicknesses at 1200 K. At the initial stage of the reaction, the volume of the Al core expands after heating, which is manifested as an increase in the thickness of the Al core. The thickness of the Al core in the first structure (a) increases by 20 Å. The expansion of the Al core in the second structure (b) is about 8 Å, and for the third structure (c) is 6 Å. That is to say, the expansion size of the Al core decreases with the increase in the thickness of the oxide layer. This is because the oxide layer will limit the thermal volume expansion of the Al core and its limiting ability increases with the increase in the thickness of the oxide layer.

At 1 ps, the Al in the structure (a) expands around the PTFE matrix. In this process, Al will immediately be defluorinated with the pyrolysis products of the PTFE matrix/PTFE, resulting in the formation of AlF_x_ on the surface of Al. When the simulation reaches about 5 ps, due to the diffusion of the PTFE matrix, the oxide layer in structures (b) and (c) is in contact with the PTFE. However, due to the blocking effect of the oxide layer, PTFE/PTFE pyrolysis products cannot directly react with cAl in the Al core. On the contrary, they react with sAl ions in the oxide layer. Because the electronegativity of F is greater than that of O, Al in Al_2_O_3_ will form AlF_x_ with F, and O in Al_2_O_3_ will diffuse to the Al core after becoming O ion and react with cAl in the Al core to regenerate cAl_2_O_3_. This reaction causes the oxide layer to move toward the Al core, which is consistent with the existing research: ‘Al_2_O_3_ catalyzes PTFE decomposition’. In addition, the defluorination reaction generates AlF_x_ on the surface of the oxide layer, which causes PTFE to lose F and form unsaturated PTFE. After the loss of F, the carbon chain of unsaturated PTFE will be exposed, and then decomposed to form small fluorocarbon molecules:

Al + PTFE = AlF_x_ + unsaturated-PTFE (1)

cAl + sAl_2_O_3_ + PTFE = sAlF_x_ + cAl_2_O_3_ + unsaturated-PTFE (2)

When the simulation reaches 10 ps, part of the PTFE carbon in structure (a) begins to deposit on the surface of Al to form Al-C clusters, and some carbon exists in the form of elemental carbon. The product AlF_x_ begins to dissociate and generate small gas molecules such as AlF_3_ and AlF₄^−^. These small molecules diffuse into the PTFE matrix and catalyze the decomposition of PTFE. In structures (b) and (c), the reaction is significantly weaker than the structure (a). At 10 ps, the oxide layer in structure (b) begins to lose its role in hindering the reaction, and the small fluorocarbon molecules begin to penetrate into the oxide layer. Additionally, the reaction is no longer confined to the outside of the oxide layer. The reaction in structure (c) still occurs outside the oxide layer. The reactions that occur in this process are as below:

AlF_x_ = AlF_3_+ AlF₄^−^ (3)

(4) $$\mathrm{PTFE}\overset{AlF_{3}}{=}{\mathrm{nC}}_{2}F_{4}$$

At 40 ps, the thickness of the product layer of structure (b) increases significantly compared with that at 10 ps, and the reaction process of Equations (3) and (4) occurs.

**Figure 9:**
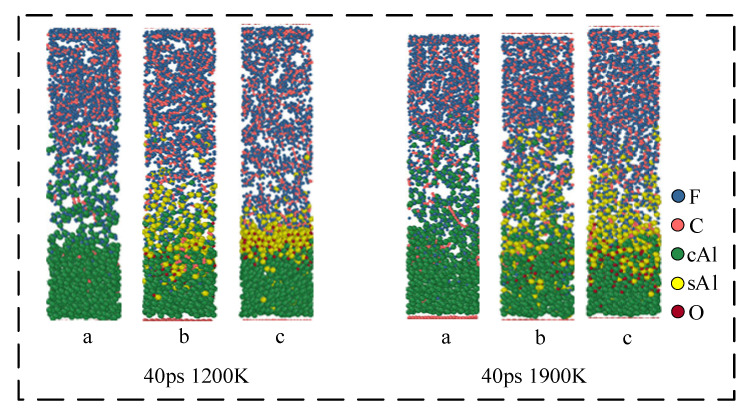
Simulation results at different temperatures at 40 ps. (a) 0 Å; (b) 5 Å; (c) 10 Å.

In Figure 9, the dynamics results of three structures at different simulation temperatures (1200 K and 1900 K) were simulated to 40 ps. The results show that in the simulation of 1200 K, no obvious carbon element is observed in structure (a). But in the simulation of 1900 K, an obvious carbon element appears in structure (a). It indicates that high temperature (1900 K) contributes to the formation of carbon elements. Under high-temperature conditions, the movement of AlF_3_ and AlF₄^−^ molecules in the system is Brownian motion. Therefore, with the increase in temperature, the diffusion degree of Al fluorine small molecules will also increase. In structure (c), the temperature will increase the moving rate of the oxide layer. When the simulation temperature is 1900 K, the oxide layer movement distance at 40 ps is significantly larger than the oxide layer movement distance at the same time when the simulation temperature is 1200 K. However, in structure (c), the reaction of Al and F still only occurs on the surface of the oxide layer.

It can be seen that under the influence of Al volume expansion, under pressure, the oxide layer contacts with PTFE and reacts. Al ions in the oxide layer react with PTFE or PTFE pyrolysis products to form AlF_x_. After a period of time, AlF_x_ is dissociated into small gas molecules such as AlF_3_ and AlF₄^−^. These small molecules diffuse into the PTFE matrix under the action of Brownian motion and catalyze the decomposition of PTFE. Due to the barrier effect of the oxide layer, fluorine-containing molecules cannot directly react with cAl in the Al core, thus forming a fluorine-rich environment outside the oxide layer. Al_2_O_3_ reacts with F ions in fluorine-containing substances to form O ions, which makes the outer surface of the oxide layer negatively charged. Under the action of the electric field, O ions diffuse into the Al core, and cAl diffuses to the oxide layer interface under the action of concentration. Under the combined action of these two atoms, the oxide layer moves toward the Al core, and cAl and sAl are exposed to the fluorine-rich environment to react. In this process, the moving rate of the oxide layer and the moving rate of the reaction interface to the center are the keys to controlling the reaction.

### 4.2. Mechanism Function

To determine the mechanism function of Al/PTFE, the α-T and dα/dT-T curves were obtained from the experimental data of simultaneous thermal analysis, where α is defined as below:

(5) $$\alpha =\frac{m_{0}-m_{t}}{m_{0}-m_{\infty }}$$

In the equation, *α* is the reaction degree, *m*_0_ is the mass at the beginning of the reaction, *m_t_* is the mass at time *t*, and *m_∞_* is the mass at the end of the reaction.

**Figure 10:**
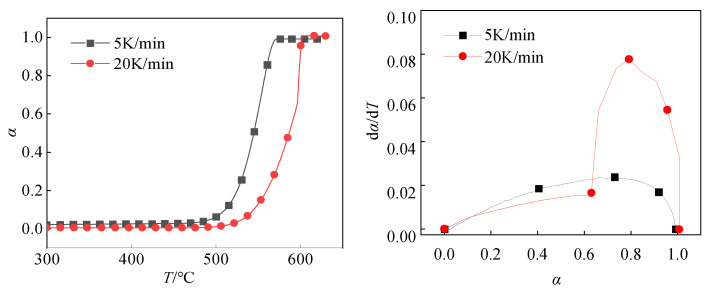
T and dα/dT-α curve.

Figure 10 shows the *α*-*T* curve and d*α*/d*T*-*T* curve with heating rates of 5 K/min and 20 K/min, respectively. When the reaction degree α does not reach 0.6, the dα/dT-α curves with heating rates of 5 K/min and 20 K/min have similar trends. It indicates that in the early stage of the reaction, the two have the same reaction process, that is, they have the same reaction mechanism function. At this time, the change trend of the two curves is similar, and the reaction rate is also close. However, when the degree of reaction reaches 0.6, the two curves are significantly different. It indicates that in the reaction process with a heating rate of 5 K/min and 20 K/min when it reaches a certain point, the reaction process with a heating rate of 20 K/min changes. In other words, the reaction mechanism function changes. This difference may be due to different heating rates leading to different dynamic behaviors of the reaction process, which in turn affects the mechanism of the reaction.

**Figure 11:**
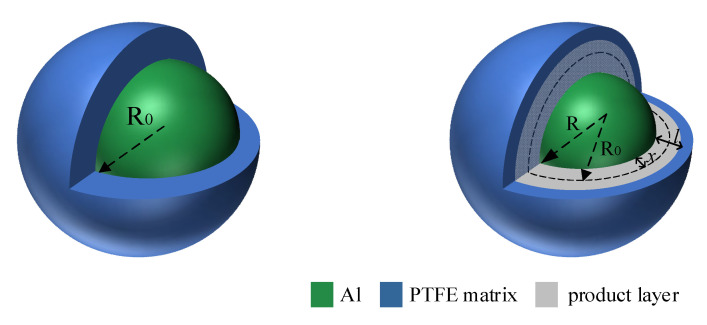
Reaction of thin oxide layer.

The mechanism function is a theoretical description of the reaction kinetic process. It can use a specific mathematical expression to describe the relationship between the rate and the reactant concentration in a specific reaction process. In the Al/PTFE molecular dynamics simulation results, it was found that the reaction rate was affected by the diffusion rate of fluorine-containing substances through the product layer in the structures with oxide layers of 0 Å and 5 Å, and the reaction process is shown in Figure 11.

When there is no oxide layer to hinder, the fluorocarbon compound diffuses to the AL core through the product layer and reacts with the Al core. *R*_0_ is the initial radius of the Al particle. *R* is the radius of the Al particle after a certain time, and *l* is the product layer. *x* is the radius of the Al particle to reduce the distance. The PTFE matrix is decomposed into fluorocarbon small molecules, and the diffusion of small fluorocarbon molecules reacts with the Al core through the product layer. The mechanism function is derived as follows.

The following equation is used to calculate the reactivity of *n* spherical particles:

(6) $$\alpha =\frac{n\frac{4}{3}\rho \pi {R_{0}}^{3}-n\frac{4}{3}\rho \pi {\left(R_{0}-x\right)}^{3}}{n\frac{4}{3}\rho \pi {R_{0}}^{3}}$$

In Equation (6), *α* is the reactivity, *R*_0_ is the initial radius of Al particles, and *x* is the reduction distance of Al particle radius. *ρ* is the Al density, and *n* is *n* Al particles in Al/PTFE. Equation (6) can be transformed into.

(7) $$\alpha =1-{\left(\frac{R_{0}-x}{R_{0}}\right)}^{3}$$

Rearrange Equation (7) to obtain:

(8) $$x=R_{0}\left[1-{\left(1-\alpha \right)}^{\frac{1}{3}}\right]$$

where *l* is the thickness of the product layer, whose growth law is Equation (9), and *k*_1_ is a rate constant. *x* and *l* satisfy Equation (10), *a* is a constant.

(9) $$l^{2}=k_{1}t$$

(10) x=al

Substitute Equations (9) and (10) into Equation (8) to obtain:

(11) $${R_{0}}^{2}{\left[1-{\left(1-\alpha \right)}^{\frac{1}{3}}\right]}^{2}=a^{2}k_{1}t$$

Assuming *k^’^*_1_ *= a*^2^*k*_1_*/R*_0_^2^, Equation (11) can be transformed into the following D3 (Jander) model.

(12) $${\left[1-{\left(1-\alpha \right)}^{\frac{1}{3}}\right]}^{2}=\frac{a^{2}k_{1}}{R^{2}}t={k_{1}}^{\prime }t$$

**Figure 12:**
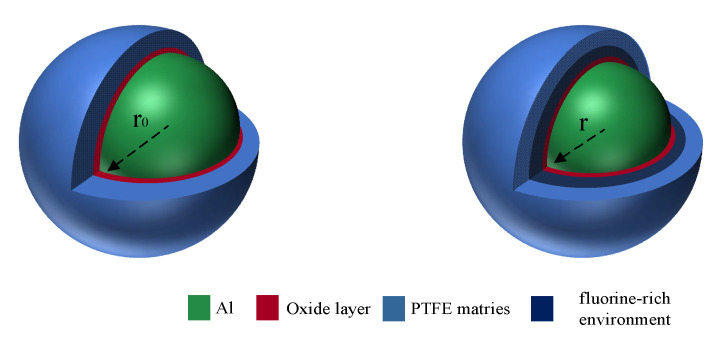
Reaction in the presence of oxide layer.

When there is an oxide layer, the reaction cannot enter the oxide layer, resulting in the accumulation of fluorine substances on the surface of the oxide layer, forming a fluorine-rich environment, as shown in Figure 12. In this case, the reaction mainly occurs on the surface of the oxide layer. Influenced by the motion of various particles, the oxide layer continues to move toward the aluminum core, and the reaction proceeds continuously.

The reactivity is calculated as follows:

(13) $$\alpha =\frac{n\frac{4}{3}\rho \pi {r_{0}}^{3}-n\frac{4}{3}\rho \pi r^{3}}{n\frac{4}{3}\rho \pi {r_{0}}^{3}}$$

Equation (13) can be simplified to Equation (14). *α* is the reaction degree, *r*_0_ is the inner radius of the initial oxide layer, and *r* is the radius of the oxide layer after a certain time. *ρ* is the Al density, and *n* is the number of Al particles in Al/PTFE. *r*_0_ and *r* satisfy Equation (15). *k*_2_ is the reaction rate constant.

(14) $$\alpha =1-\frac{r^{3}}{{r_{0}}^{3}}$$

(15) $$r=r_{0}-k_{2}t$$

Substituting Equation (15) into Equation (14), the following equation can be obtained:

(16) $$\alpha =1-{\left(\frac{r_{0}-k_{2}t}{r_{0}}\right)}^{3}$$

After rearranging Equation (16), we obtain:

(17) $$1-\alpha ={\left(1-\frac{k_{2}}{r_{0}}t\right)}^{3}$$

Let *k*_2_*’ = k*_2_*/r*_0_, Equation (17) becomes as below.

(18) $$1-{\left(1-\alpha \right)}^{\frac{1}{3}}={k_{2}}^{\prime }t$$

Equation (18) is the R3 model. Combined with the α-T curve, dα/dT-T curve, and kinetic results obtained by the DSC experiment, it can be seen that in the actual process, the initial Al/PTFE reaction process is hindered by the oxide layer. The reaction only reacts outside the oxide layer. The mechanism function is R3 at first. At a certain time, the oxide layer loses its hindrance, and the reaction diffuses into the oxide layer. The reaction mechanism function of Al/PTFE is transformed into D3, so the reaction mechanism function of Al/PTFE can be expressed as follows:

(19) $$f(\alpha )=\left\{\begin{array}{lll} 3{\left(1-\alpha \right)}^{\frac{2}{3}} & \left(R3\right) & \alpha <\alpha_{c}\\ \frac{3}{2}{\left(1-\alpha \right)}^{\frac{2}{3}}{\left[1-{\left(1-\alpha \right)}^{\frac{1}{3}}\right]}^{-1} & \left(D3\right) & \alpha >\alpha_{c} \end{array}\right.$$

where 0 ≤ *α_c_* ≤ 1. *α_c_* will change with the heating rate, ignition mode, and other reactive materials in different environments. In the TG-DSC experiment with a heating rate of 5 K/min, *α_c_* is 1. Additionally, in the TG-DSC experiment with a heating rate of 20 K/min, *α_c_* is 0.6.

### 4.3. Parameters of Rate Equation

For chemical reactions, the reaction rate can be described as follows:

(20) $$\frac{d\alpha }{dt}=Ae^{-(\frac{E_{a}}{RT})}f(\alpha )$$

The apparent activation energy (*E*_a_) in Equation (20) is the threshold or energy barrier that must be overcome in the occurrence of chemical bond recombination, which intuitively reflects the difficulty of a system reaction. The lower the apparent activation energy is, the easier the reaction is. On the contrary, the higher the apparent activation energy is, the more difficult the reaction is. *A* is a pre-exponential factor, which is a measurement of the frequency of the reaction. The reaction activation energy and pre-exponential factor of the Al/PTFE system can be calculated according to the temperature *T*_p_ (Table 2) and Kissinger equation (Equation (21)) corresponding to the exothermic peak at different heating rates in the TG-DSC curve.

(21) $$\ln\frac{\beta_{i}}{T_{pi}^{2}}=\ln\frac{R\cdot A}{{Ε}_{a}}-\frac{{Ε}_{a}}{R\cdot T_{pi}},\;i=1,2,3,4$$

*β_i_* is the heating rate (K/min); *T*_pi_ is the peak temperature of the DSC curve (K); *E_a_* is the activation energy (KJ/mol); *R* is the gas constant, 0.008314 KJ/(mol·K); *A* is the pre-exponential factor (min^−1^).

**Figure 13:**
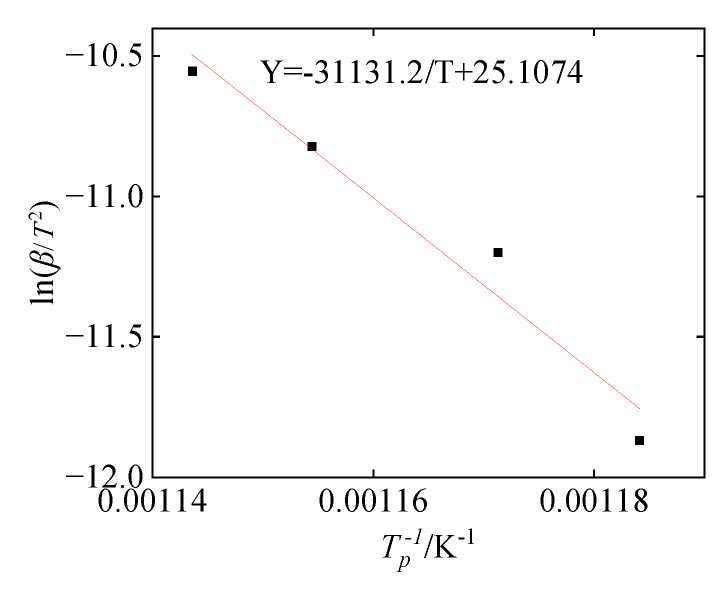
Arrhenius curve by Kissinger fitting.

The Kissinger equation shows that 1*/T*_pi_ has a linear relationship with ln (*β_i_/T*_pi_^2^), and a straight line can be obtained by plotting the two. The activation energy *E*_a_ and pre-exponential factor *A* of the system reaction can be calculated by the slope and intercept after linear fitting. Figure 13 shows the Arrhenius curve by Kissinger fitting, where the slope is *E*_a_*/R*, the value is 31,131.2, and the calculated activation energy *E*_a_ is about 258.8 KJ/mol. The intercept is ln(*R·A/E*_a_), the value is 25.1074, and the calculated pre-exponential factor *A* is 2.495 × 10^15^ min^−1^. According to the experimental parameters and the *f*(*α*) obtained in Section 4.2, the chemical reaction rate can be expressed as below:

(22) $$\frac{d\alpha }{dt}=\left\{\begin{array}{lll} 2.495\times 10^{12}e^{-(\frac{258}{RT})}3{\left(1-\alpha \right)}^{\frac{2}{3}} & R3 & \alpha <\alpha_{c}\\ 2.495\times 10^{12}e^{-(\frac{258}{RT})}\frac{3}{2}{\left(1-\alpha \right)}^{\frac{2}{3}}{\left[1-{\left(1-\alpha \right)}^{\frac{1}{3}}\right]}^{-1} & D3 & \alpha >\alpha_{c} \end{array}\right.$$

**Figure 14:**
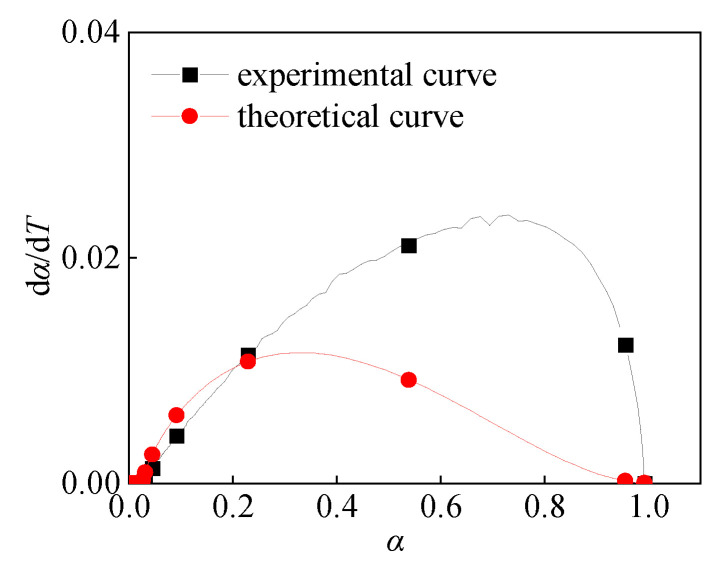
d*α*/d*T*-*α* Curve at 5 K/min.

There are two different mechanism functions in the continuous *α* in the Al/PTFE reaction process. The range of *α_c_* is: 0 ≤ *α*_c_ ≤ 1, and the value of *α_c_* is related to the environment of Al/PTFE (heating rate, ignition mode, impact velocity, etc.). When the TG-DSC experiment of Al/PTFE was conducted at a heating rate of 5 K/min, α_c_ = 1, the reaction mechanism function in the whole reaction process was R3. Using the following Equation (23) to calculate the curve of dα/d*T* and α (Figure 14), the theoretical curve and the experimental curve have a high degree of fitting.

(23) $$\frac{d\alpha }{dT}=\frac{d\alpha }{dt}\cdot \frac{dt}{dT}=\frac{A}{\beta }\cdot e^{-(\frac{E_{a}}{RT})}\cdot 3{\left(1-\alpha \right)}^{\frac{2}{3}}$$

### 4.4. Effect of Oxide Layer Thickness on Activation Energy

To study the effect of oxide layer thickness on the reaction behavior of Al/PTFE, reactive molecular dynamics simulation was implemented to calculate the apparent activation energy *E_a_* of three structures (a, b, and c). In structures (a) and (b), the reaction mechanism function adopts the D3 model. The parabolic law equation (Equation (9)) is used to calculate the reaction rate constant *k*_1_ at different simulated temperatures. Specifically, the change of the product layer thickness *l* with the reaction time *t* during the reaction process was observed, and *k*_1_ was obtained by parabolic law fitting. The kinetic model of structure c is the R3 model, and the reaction rate constant *k*_2_ was calculated by using Equation (15) and dynamics results.

**Figure 15:**
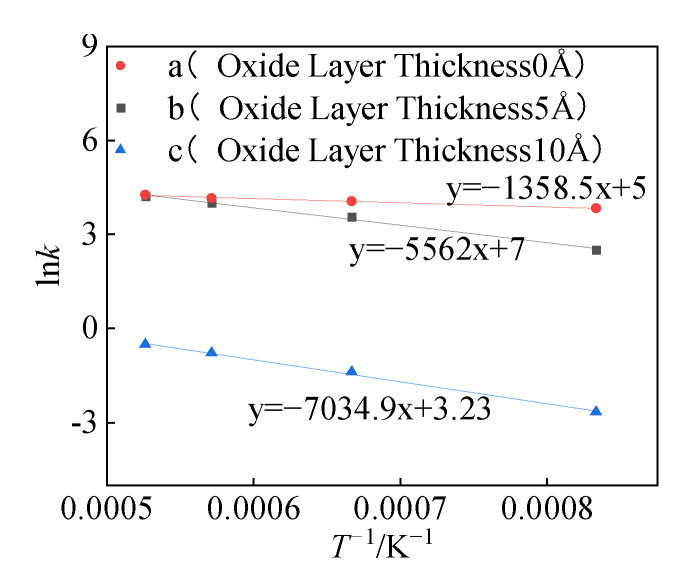
Arrhenius curve.

*E*_a_ was calculated by the Arrhenius equation (Equation (24)) and *k* (*k*_1_, *k*_2_) was obtained at different temperatures of structures (a, b, and c). Among them, *k*_1_ and *k*_2_ have a certain proportional relationship with *k’*_1_ and *k’*_2_ in the reaction mechanism function model, which will not affect the slope (*E*_a_*/R*) in the Arrhenius curve. Therefore, the activation energy of the structure can be obtained by linear fitting using *k*_1_ and *k*_2_, as shown in Figure 15.

(24) $$\ln k=\ln A-\frac{E_{a}}{RT}$$

**Figure 16:**
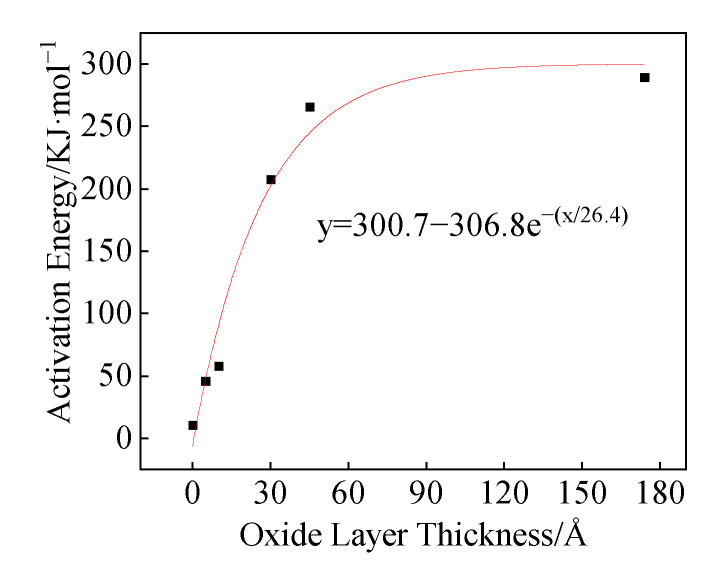
*E*_a_ changes upon oxide layer thickness change.

It can be seen from Figure 16 that the activation energies of the three structures (a, b, and c) are 11.3 KJ/mol, 46 KJ/mol, and 58 KJ/mol, respectively. Zeng et al. obtained the relationship between the Al particle size and the thickness of the oxide layer [24,26]:

(25) $$\delta =\left[3.621\left(1-e^{-0.0248d}\right)+1.251\left(1-e^{-0.0012d}\right)\right]$$

(26) $$\delta =24.705(1-e^{-0.409d})$$

In the equation, δ is the thickness of the oxide layer and d is the particle size of the Al particles. The oxide layer of the Al particle size used in this paper is calculated to be about 3.5 nm by using Equation (25). When the activation energy is 258.8 KJ/mol, the oxide layer thickness is 5.3 nm, indicating that Equations (25) and (26) have high rationality.

Although the reaction mechanism functions of structures (a) and (b) are consistent, the presence or absence of the oxide layer has a significant effect on the activation energy. In structure (b), the existence of the oxide layer increases the activation energy from 11.3 KJ/mol to 46 KJ/mol. The oxide layer cannot hinder the diffusion of fluorocarbon molecules into the oxide layer and reach the Al core during the reaction process. However, compared with structure (a) without the oxide layer, the oxide layer in structure (b) will affect the diffusion coefficient D of fluorocarbon molecules in the product layer, which in turn affects the activation energy. The diffusion coefficient D can be calculated by the following Equation (27):

(27) $$D=\frac{k_{1}}{2}\rho$$

In the equation, *k_1_* is the rate constant, and *ρ* is the density of fluorocarbon molecules. The calculation shows that the diffusion coefficient D of structure (a) at 1200 K is 3.66109 m^2^/s, and the diffusion coefficient D at 1900 K is 5.6109 m^2^/s. The diffusion coefficient of structure (b) at 1200 K is 9.8108 m^2^/s, and the diffusion coefficient at 1900 K is 5.4109 m^2^/s. When the simulation temperature is 1200 K, the diffusion coefficient is significantly different. At 1900 K, the 5 Å oxide layer basically loses its effect, and the diffusion coefficients of the two are close. The difference in diffusion coefficients in different structures affects the activation energy of the reaction.

By increasing the thickness of the oxide layer from 5 Å to 10 Å (structure from b to c), the reaction process changed significantly. Different from the oxide layer in structure (b), which is rearranged, and the compactness decreases during the thermal expansion process, the oxide layer in structure (c) maintains compactness throughout the reaction process. Increasing the thickness of the oxide layer can increase the compactness of the oxide layer so that the oxide layer does not appear defects during the expansion process. So, the activation energy of structure (c) is greater than the activation energy of structure (b).

## 5. Conclusions

In this paper, Al/PTFE reactive materials with Al particle sizes at the nanometer level were prepared by wet mixing. The TG-DSC experiment was carried out to test the thermochemical reaction of the samples at different heating rates. The Al/PTFE reaction process with different oxide layer thicknesses was studied by ReaxFF reaction molecular dynamics. The following main conclusions are drawn.

The effects of temperature and heating rate on the reaction process are significant. High temperature contributes to the formation of carbon, and temperature has a significant effect on the movement of small gases such as molecules of AlF_3_ and AlF₄^−^ and the movement rate of the oxide layer in the structure. As the heating rate increases, the thermal effect per unit time increases, the thermal inertia increases, and the temperature difference increases. As a result, the reaction exothermic peak and other characteristic temperatures move to the high-temperature direction.

The thickness of the oxide layer has a significant effect on the reaction process of Al/PTFE. When the oxide layer is thin, the compactness of the oxide layer changes due to thermal rearrangement, resulting in the diffusion of reactants (fluorine-containing substances) through the oxide layer into the Al core. Additionally, the reaction mainly occurs between the oxide layer and the Al core. When the oxide layer is thick, the reaction only exists outside the oxide layer interface. With the movement of oxygen ions in the oxide layer and Al atoms in the Al core, the oxide layer moves to the Al core, so that the reaction continues. In addition, a pre-ignition reaction will occur between the oxide layer and the PTFE matrix, which will catalyze the decomposition of PTFE and further affect the reaction process.

The mechanism function of Al/PTFE is composed of the R3 model and the D3 model. The critical value *α_c_* depends on the reaction environment such as ignition mode, impact velocity, and heating rate.

## Figures and Tables

**Table 1:** TG-DSC experimental conditions.

Term	Condition
Temperature °C	30–800
Heating Rate K/min	5, 10, 15, 20
Sweeping Gas	Ar
Sample Mass mg	10 ± 0.5
Reference	Al_2_O_3_
Crucible	Al_2_O_3_

**Table 2:** Characteristic temperature of thermal analysis.

Ingredient	Heating Rate K/min	*T*_i_/°C	*T*_p_/°C	*T*_f_/°C	Energy Release J/g	Mass Loss %
Al/PTFE	5	539.8	572.4	592.8	260	63.1
Al/PTFE	10	561.34	580.6	607.3	240	92
Al/PTFE	15	569.4	593.1	666.4	229	91.5
Al/PTFE	20	591	601	633	316	90.9

**Table 3:** Simulation conditions.

No.	Layer	Simulation Temperature	Number of PTFE
a	0 Å	1200 K, 1500 K, 1750 K, 1900 K	42
b	5 Å	1200 K, 1500 K, 1750 K, 1900 K	42
c	10 Å	1200 K, 1500 K, 1750 K, 1900 K	42

## Data Availability

The original contributions presented in the study are included in the article, further inquiries can be directed to the corresponding authors.

## References

[1] Wang H., Zheng Y., Yu Q., Liu Z., Yu W. (2011). Impact-induced initiation and energy release behavior of reactive materials. J. Appl. Phys..

[2] Zheng Y., Su C., Guo H., Yu Q., Wang H. (2021). Chain damage effects of multi-spaced plates by reactive jet impact. Def. Technol..

[3] Daniels A.S., Baker E.L., DeFisher S.E., Ng K.W., Pham J. Bam bam: Large scale unitary demolition warheads. Proceedings of the 23rd International Symposium on Ballistics.

[4] Wang H.F., Xiang J.A. (2023). Progress in reactive materials and their applications. Sci. Sin. Tech..

[5] Yang X., He Y., He Y., Wang C., Zhou J., Xu T. (2020). 3D Meso-scale Simulation on the Shock Compression Behaviors of Al/PTFE Reactive Materials Based on the CT Slices. Chin. J. Exphosives Propellants (Huozhayao Xuebao).

[6] Cai S., Jiang C., Mao L., Wang Z., Hu R., Ye S. (2021). Impact Temperature Rise Law of Porous Aluminum-rich PTFE/Al Energetic Material. Acta Armamentarii.

[7] Lu G., Li P., Liu Z., Xie J., Ge C., Wang H. (2022). Theoretical model for the impact initiated chemical reaction of AL/PTFE reactive material. Materials.

[8] Levitas V.I. (2013). Mechanochemical mechanism for reaction of aluminium nano-and micrometre-scale particles. Philos. Trans. R. Soc. A Math. Phys. Eng. Sci..

[9] Losada M., Chaudhuri S. (2009). Theoretical study of elementary steps in the reactions between aluminum and teflon fragments under combustive environments. J. Phys. Chem. A.

[10] Tao J., Wang X. (2023). Experimental study and simulation of the reaction mechanism of Al–PTFE mechanically activated energetic composites. RSC Adv..

[11] Rosencrantz S.D. (2007). Characterization and Modeling Methodology of Polytetrafluoroethylene Based Reactive Materials for the Development of Parametric Models.

[12] Ren S., Zhang Q., Wu Q., Long R., Liang H., Gong L. (2019). A debris cloud model for hypervelocity impact of the spherical projectile on reactive material bumper composed of polytetrafluoroethylene and aluminum. Int. J. Impact Eng..

[13] Zhou J. (2018). Study on the Impact-Induced Reaction Characteristics of Typical Fluoropolymer-Matrix Reactive Materials.

[14] Zheng Y.-F., Zheng Z.-J., Lu G.-C., Wang H.-F., Guo H.-G. (2023). Mesoscale study on explosion-induced formation and thermochemical response of PTFE/Al granular jet. Def. Technol..

[15] Tian W., He Y., Wang C., Guo L., Zhou J. (2022). Heterogeneous chemical reaction model of Al/PTFE reactive materials under impact load. J. Nanjing Univ. Sci. Technol..

[16] Mao L., Ye S., Hu W., Jiang C., Wang Z. (2020). Thermochemical Reaction Characteristics of PTFE/Al Reactive Material. Acta Armamentarii.

[17] Gao Y., Zhu W., Wang T., Yilmaz D.E., van Duin A.C.T. (2022). C/H/O/F/Al ReaxFF Force Field Development and Application to Study the Condensed-Phase Poly (vinylidene fluoride) and Reaction Mechanisms with Al. J. Phys. Chem. C.

[18] Van Duin A.C.T., Dasgupta S., Lorant F., Goddard W.A. (2001). ReaxFF: A reactive force field for hydrocarbons. J. Phys. Chem. A.

[19] Chenoweth K., Van Duin A.C.T., Goddard W.A. (2008). ReaxFF reactive force field for molecular dynamics simulations of hydrocarbon oxidation. J. Phys. Chem. A.

[20] Senftle T.P., Hong S., Islam M., Kylasa S.B., Zheng Y., Shin Y.K., Junkermeier C., Engel-Herbert R., Janik M.J., Aktulga H.M. (2016). The ReaxFF reactive force-field: Development, applications, and future directions. npj Comput. Mater..

[21] Senftle T.P., Meyer R.J., Janik M.J., van Duin A.C.T. (2013). Development of a ReaxFF potential for Pd/O and application to palladium oxide formation. J. Chem. Phys..

[22] Liu J., Xiong K., Zhang H., Nie H., Yan Q.-L. (2023). The effect of alumina as an interfacial layer on the reactivity of Al/PTFE energetic composites. J. Mater. Res. Technol..

[23] Mock W., Drotar J.T. (2007). Effect of aluminum particle size on the impact initiation of pressed PTFE/Al composite rod. AIP Conf. Proc..

[24] Zeng L., Jiao Q.J., Ren H., Zhou Q. (2011). Effect of particle size of nano-aluminum powder on oxide film thickness and active aluminum content. Chin. J. Explos. Propellants.

[25] Thompson A.P., Aktulga H.M., Berger R., Bolintineanu D.S., Brown W.M., Crozier P.S., in ‘t Veld P.J., Kohlmeyer A., Moore S.G., Nguyen T.D. (2022). LAMMPS—A flexible simulation tool for particle-based materials modeling at the atomic, meso, and continuum scales. Comput. Phys. Commun..

[26] Zeng L., Jiao Q., Ren H., Zhou Q. (2012). Studies on oxide film thickness and activity of micron aluminum powder. Trans. Beijing Inst. Technol..

